# Biochemical Applications of Microbial Rare Glycan Biosynthesis, Recognition, and Sequencing

**DOI:** 10.1021/acs.biochem.5c00338

**Published:** 2025-08-20

**Authors:** Joanna Joo, Andrea Koid, Hanee Kim, Antara Ghosh, Seayoung Lee, Mia Sheshova, Tania J. Lupoli

**Affiliations:** Department of Chemistry, 5894 New York University, New York, New York 10003, United States

## Abstract

While humans utilize approximately ten building blocks, hundreds of “rare” sugars exist, which are absent in mammals but present in microbes, plants, and other natural sources. In addition to the common sugars found across organisms, more than 700 different rare monosaccharides exist, many of which are prokaryote-specific and utilized across bacteria to decorate natural products and various other glycoconjugates. As the outer glycocalyx layer of bacterial cells is composed of glycolipids, glycoproteins, and polysaccharides, rare sugars are enriched on the cell surface and are major components of structures known to mediate interactions with other cells and the environment. Despite their importance in biology, there remain many open questions in the field of biochemistry regarding the biosynthesis and functions of rare sugars. This perspective highlights ongoing biochemical work on prokaryotic rare sugars, including approaches to study the incorporation of rare sugars into cellular glycans, to develop chemical and enzymatic routes for generating rare sugar probes and glycans, and to analyze rare sugar–protein interactions. Opportunities to improve the sequencing efforts of microbial glycans through experimental and computational approaches are also discussed, along with potential therapeutic applications of rare sugar-containing molecules. In covering these topics, we emphasize tools that have not yet been utilized to study rare sugars but may be used for future approaches that will expand our knowledge of their distinct roles in microbes and the interplay between pathogens and their hosts.

## Graphical abstract

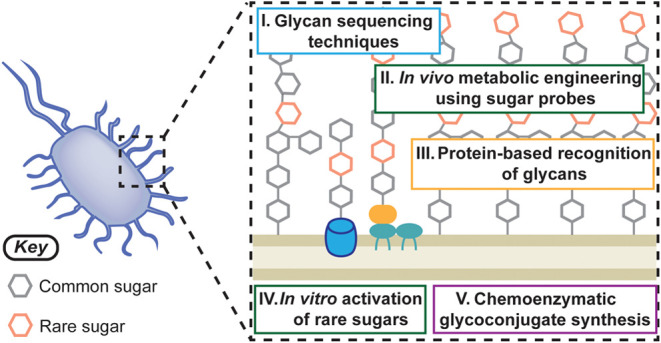

## Introduction

Carbohydrates are the most abundant biomolecules on Earth. While approximately ten “common” monosaccharide building blocks are conserved across species, there exists a wealth of unique monosaccharide structures absent in mammals, termed “rare,” and largely produced in plants, lower eukaryotes, and prokaryotes. The Carbohydrate Structure Database (CSDB) contains >700 naturally occurring sugars,^1,2^ the majority of which are produced by microbes and termed “prokaryote-specific”.^3^ Other definitions for sugar classes also exist, as the International Society of Rare Sugars categorizes only seven sugars (d-glucose (d-Glc), d-mannose (d-Man), d-xylose (d-Xyl), d-galactose (d-Gal), d-ribose, d-fructose, and l-arabinose) as abundant enough to be considered “common”.^3,4^ However, “rare” might serve as a misnomer for many of the remaining sugars, as they can exist in high quantities in plants, fungi, bacteria, archaea, and the many molecules secreted by these organisms.^3–5^ For instance, l-rhamnose (l-Rha) is among the most prevalent of the rare sugars found in nature, as it is enriched in the surface and structural glycans of plants and bacteria, in addition to serving as a functional group for natural product derivation.^6^ Rare glycans are essential in some bacterial species and play other important roles in biology, as indicated by their presence in molecules used for food, agriculture, and medical applications.^7^ Accordingly, obtaining a biochemical understanding of the biosynthesis, assembly, and recognition of rare sugars is an active area of research (Figure 1).

**Figure 1:**
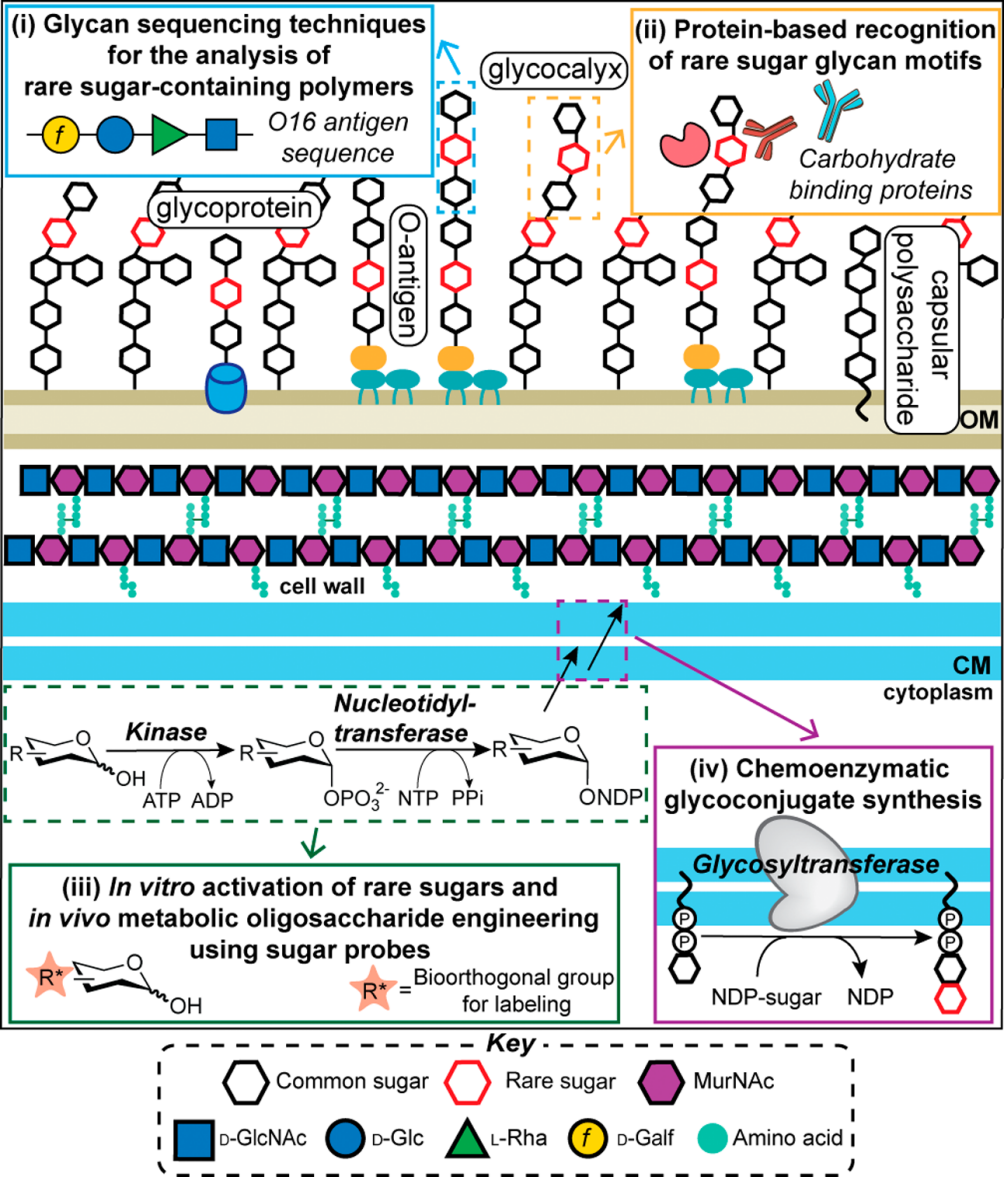
Rare sugars are present in many bacterial glycans. Overview of biochemical approaches currently used to study the following: (i) sequences of rare sugars and glycans, (ii) recognition of rare sugar-containing glycan motifs, (iii) activation of rare sugars, and (iv) their incorporation into glycans for the biosynthesis of glycoconjugates containing rare sugars. Note that MurNAc, l-Rha, and Gal*f* are all rare sugars. OM, outer membrane; CM, cytoplasmic membrane.

Glycome analyses have revealed that the rare sugars of microbes are structurally distinct from the structures of mammalian sugars. In particular, bacterial rare monosaccharides are enriched in 6-deoxy sugars that lack a hydroxyl at the C(6) position, as well as l- as opposed to d-sugars, and furanose and heptose sugars that are not found in mammals.^8^ Many glycans across cell types are enzymatically assembled from both common and rare monosaccharide building blocks that are typically phosphorylated and then activated as (deoxy)­nucleoside diphosphate sugars ((d)­NDP sugars) (Figure 1, box (iii)). While mammalian sugars are often activated as uridine diphosphate (UDP) or guanosine diphosphate (GDP) sugars, many microbial sugars are activated as deoxythymidine diphosphate (dTDP) conjugates, in addition to other (d)­NDP-activating groups.^9^ Dedicated glycosyltransferases utilize these nucleotide sugars as donor substrates for transfer to biomolecular acceptors to build an incredible diversity of glycoconjugates (Figure 1, box (iv)), including glycolipids that serve as precursors for the structural components of bacterial cell envelopes.^3^ These envelopes consist of the cytoplasmic membrane, peptidoglycan (cell wall) layer, and an additional outer membrane found in Gram-negative bacteria and and some other bacteria, including mycobacteria.^10^

The bacterial glycocalyx, which constitutes the outermost layer of the cell envelope, contains rare sugars within various glycan structures. In Gram-negative bacteria, the outer leaflet of the outer membrane is composed of lipopolysaccharide (LPS), which is known to be antigenic.^11^ LPS contains the anchoring glycolipid Lipid A, or endotoxin, linked to core oligosaccharides, which can be attached to a polysaccharide, called O-antigen (O-Ag), made up of repeating oligosaccharide units (O-units). The presence of Lipid A attached to two residues of the rare sugar 3-deoxy-d-*manno*-oct-2-ulosonic acid (Kdo) is essential for the growth of Gram-negative bacteria under laboratory conditions.^12^ While other rare sugars are present in the core oligosaccharides, the greatest diversity of rare sugars is found in the O-Ag. Across the well-studied Gram-negative bacterium*Escherichia coli* alone, >180 different serotypes can be distinguished by different sequences of >20 different monosaccharides found within expressed O-units (Figure 1, box (i)).^13^ About half of the monosaccharide building blocks across the O-Ag are rare sugars. Many microbial species that are human pathogens, including those from *Salmonella*, *Klebsiella*, and *Shigella*, produce O-Ag’s containing rare sugars.^11^ Other bacterial surface glycans also contain repeating sequences of rare and common sugars that are used for serotyping, such as Gram-negative and -positive capsular polysaccharides.^14,15^ In pathogens, many of these polymers mediate interactions with the host and act as virulence factors (Figure 1, box (ii));^16,17^ further, repeating glycan sequences have recently served as antigens for the development of vaccines.^18^ Hence, there has been a growing interest in developing concise chemical routes to microbial glycans and improved access to enzymatic precursors.^19,20^

In this perspective, we provide context for our current knowledge of the biochemistry of rare sugars and highlight work performed in the last 5 years relevant to both chemical and chemoenzymatic syntheses of activated rare sugars and glycans, the detection of surface-exposed rare glycans with proteins, and examples of new technologies in rare glycan sequencing (Figure 1). Much work in this area has focused on the biochemistry and chemical biology of peptidoglycan synthesis and degradation across bacteria, as the cell wall contains a conserved glycan backbone composed of *N*-acetylglucosamine (GlcNAc) and the rare sugar *N*-acetyl muramic acid (MurNAc). However, this research has been expertly reviewed elsewhere.^21,22^ Here, we focus on other rare sugar-containing glycoconjugates and primarily those found in bacteria. Due to the chemical diversity of rare sugars and the complexity of microbial glycan structures, we discuss the tremendous opportunities for biochemists to characterize enzymes involved in the assembly and recycling of these structures, along with the discovery of new carbohydrate-binding proteins (CBPs) that may aid in rare glycan recognition and sequence determination.

### Metabolic Oligosaccharide Engineering (MOE) Using Bacterial Sugar Probes Highlights Promiscuity within Glycan Biosynthetic Pathways

Metabolic oligosaccharide engineering (MOE) is a widely used method for labeling glycans in different cell types and has enabled the identification of novel cell–cell interactions via cross-linking and other chemical biology approaches.^3,21,22^ Often, bioorthogonal functional groups, such as azides, are added to sugar scaffolds, and cellular pathways incorporate these sugar analogues into glycans, which are then detected using orthogonal reactions with reporter molecules (Figure 2A). While this approach was first validated in eukaryotic cells by Bertozzi and co-workers,^23,24^ more recent examples include utilization of synthetic bacterial sugar analogues for the labeling of bacterial glycans in live cells.^22^ By evaluating which glycans are modified by a particular sugar probe, researchers are able to broadly evaluate the following stages of glycan biosynthesis: (i) activation of unnatural sugars to form the corresponding nucleotide sugar, (ii) transfer of the unnatural sugar into a growing glycan, and (iii) transport of that unnatural glycan to its final destination in the cell. Hence, probe usage offers us insight into the substrate promiscuity of many glycan-processing enzymes at once, in addition to providing bioorthogonal handles to perform chemical reactions on biomolecules.

**Figure 2:**
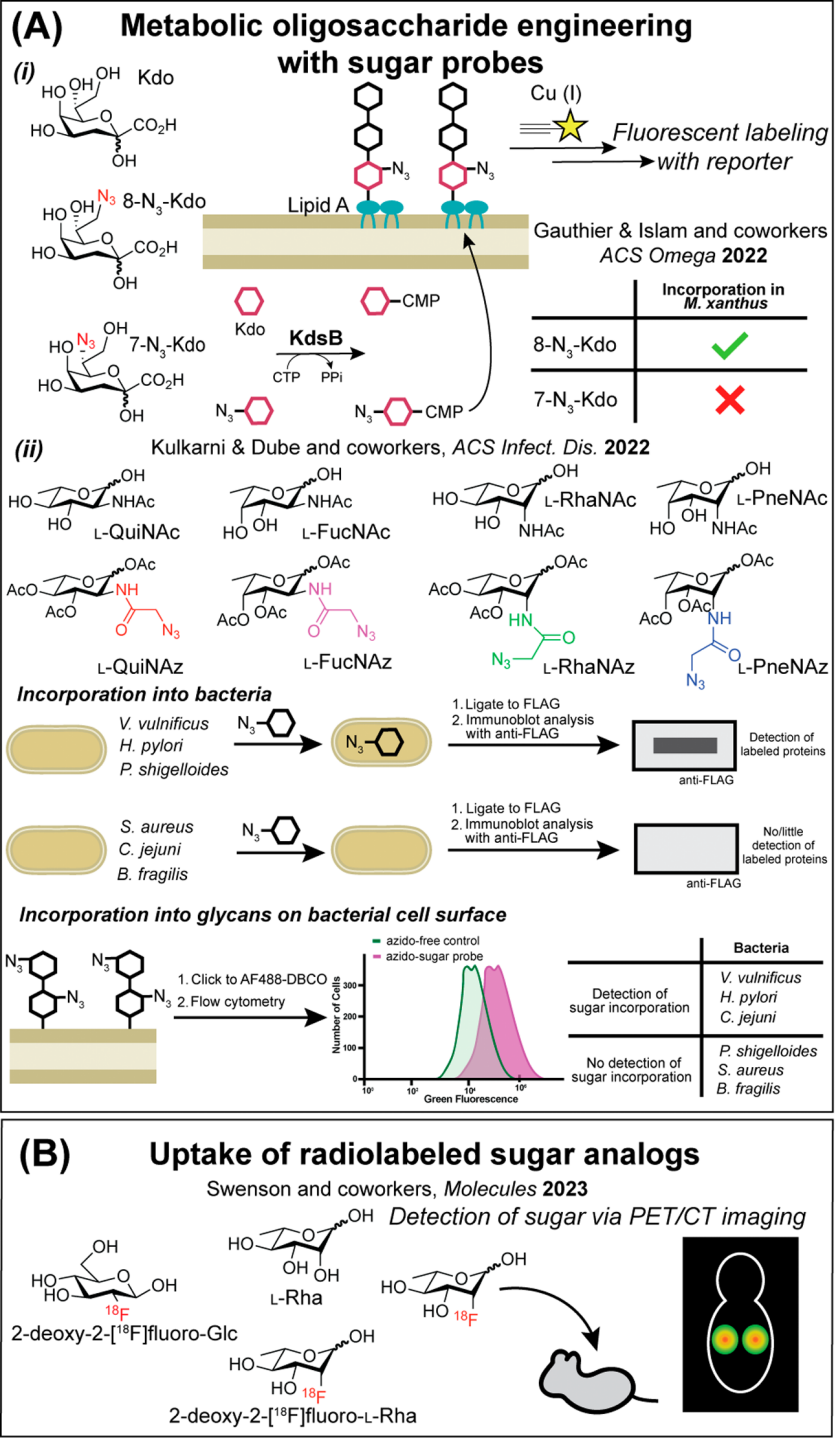
Metabolic oligosaccharide engineering (MOE) using rare sugars provides insight into relevant biochemical pathways. (A) (i) Bioorthogonal Kdo probes used to study labeling of LPS in Gram-negative bacteria. (ii) Rare sugar probes with bioorthogonal handles synthesized and used for incorporation analysis into different glycan components of various bacterial species. Schematic examples of immunoblot and fluorescence-activated cell sorting (FACS) results with different bacteria are shown (dibenzocyclooctyne is DBCO). (B) Analysis of synthetic sugars indicated that a ^18^F-labeled l-Rha analogue could be detected using positron emission tomography/computed tomography (PET/CT) imaging in mice as a future method to label sites of infection in a host.

Some of the most extensive MOE work across bacterial species has taken advantage of Kdo analogues to label LPS in Gram-negative bacteria. Activation of Kdo occurs via the addition of cytidine monophosphate (CMP) from cytidine triphosphate (CTP) to the sugar, catalyzed by the CMP-Kdo synthetase KdsB (Figure 2A, (i)).^25^ This direct enzymatic activation of Kdo monosaccharides can be leveraged, as structural analogues of Kdo fed into the cell are converted into nucleotide sugars without the need for separate kinase and nucleotidyltransferase activities (see Figure 1). A bioorthogonal Kdo probe, 8-azido-8-deoxy-Kdo (8-N_3_-Kdo), was first synthesized in 2012 and shown to label various Gram-negative bacteria by the Dukan and Vauzeilles groups, followed by others,^25–27^ using Cu­(I)-catalyzed azide–alkyne cycloaddition (CuAAC) and strain-promoted azide–alkyne cycloaddition (SPAAC) reactions with reporters. However, direct replacement of native Kdo residues with the azido analogue was not validated until 2017 by Nilsson and co-workers after extensive characterization of purified modified *E. coli* LPS.^28^ This analysis revealed that adding 8-N_3_-Kdo to Kdo-deficient cells led to truncated LPS cores in addition to full-length cores. Further, *E. coli* KdsB was shown to have a 6.5-fold higher *K*_M_ for 8-N_3_-Kdo than its native Kdo substrate.^28^ It should be noted that, decades ago, Raetz and co-workers found that the glycosyltransferase KdtA (WaaA) must transfer two Kdo residues onto Lipid IV, the precursor of Lipid A,^29^ for complete synthesis of the inner core to occur; however, only one sugar is transferred when CMP-Kdo levels are submillimolar.^30^ Hence, Nilsson and co-workers hypothesized that low levels of CMP-8-N_3_-Kdo may lead to the observed truncation of the LPS inner core due to only a single addition of 8-N_3_-Kdo residues. Further analysis of the substrate scope of KdtA would be needed to fully validate this postulation. Notably, differences in the ability of 8-N_3_-Kdo to label cell surfaces across Gram-negative bacteria have been attributed to the required presence of the sialic acid transporter NanT for uptake of Kdo probes.^28^ Namely, *E. coli* and *Klebsiella pneumoniae* contain this transporter, while *Pseudomonas aeruginosa* does not; thus, the latter’s outer membrane is not modified by azido-Kdo probes.^31^

Work performed in 2022 by the Gauthier and Islam groups compared 8-N_3_-Kdo to 7-azido-7-deoxy-Kdo (7-N_3_-Kdo) for incorporation into the LPS of *Myxococcus xanthus* to probe outer membrane exchange and vesicle mechanisms involved in swarming behavior (Figure 2A, (i)).^32^ Analysis of *M. xanthus* cells after incubation with each probe revealed that while 8-N_3_-Kdo incorporation could be detected on the cell surface, that of 7-N_3_-Kdo could not.^32^ Instead, the latter appears to be catabolized by these bacteria, as both probes were hypothesized to be substrates for at least one of the several putative sialic acid transporters. Biochemical analysis of *E. coli* KdsB again showed an increase in the *K*_M_ for 8-N_3_-Kdo compared to Kdo, but even millimolar concentrations of 7-N_3_-Kdo were not consumed by the enzyme. Hence, this work demonstrates that the active site does not accommodate modification at position C(7) compared to the C(8) position of Kdo. Several crystal structures of KdsB have been reported, and a cocomplex of *E. coli* KdsB bound to CTP and a Kdo analogue illustrated that the active site can accommodate modification of the C(8) position, but bulky groups would be sterically occluded by residues that make contacts with the C(7) position.^33^ Hence, this current work supports the hypothesis that KdsB represents a “bottleneck” for the incorporation of Kdo analogues into cells. Due to the high cost of commercial 8-N_3_-Kdo, it should be noted that Gauthier, Islam, and co-workers improved the production of this probe appreciably compared to previous reports via optimization of the synthesis of an intermediate to yield gram amounts of 8-N_3_-Kdo, along with a route to the novel analogue 7-N_3_-Kdo, both via a Cornforth homologation procedure.^32,34^ As depicted in Scheme 1, commercially available d-arabinose and l-xylose underwent Fischer glycosylation followed by a regioselective tosylation to achieve intermediates (**1,2**) and nucleophilic substitution followed by Cornforth homologation to generate the target molecules. Hence, this updated route may offer easier access to Kdo analogues for the continued analysis of this essential LPS core oligosaccharide biosynthetic pathway.

**Scheme 1:**
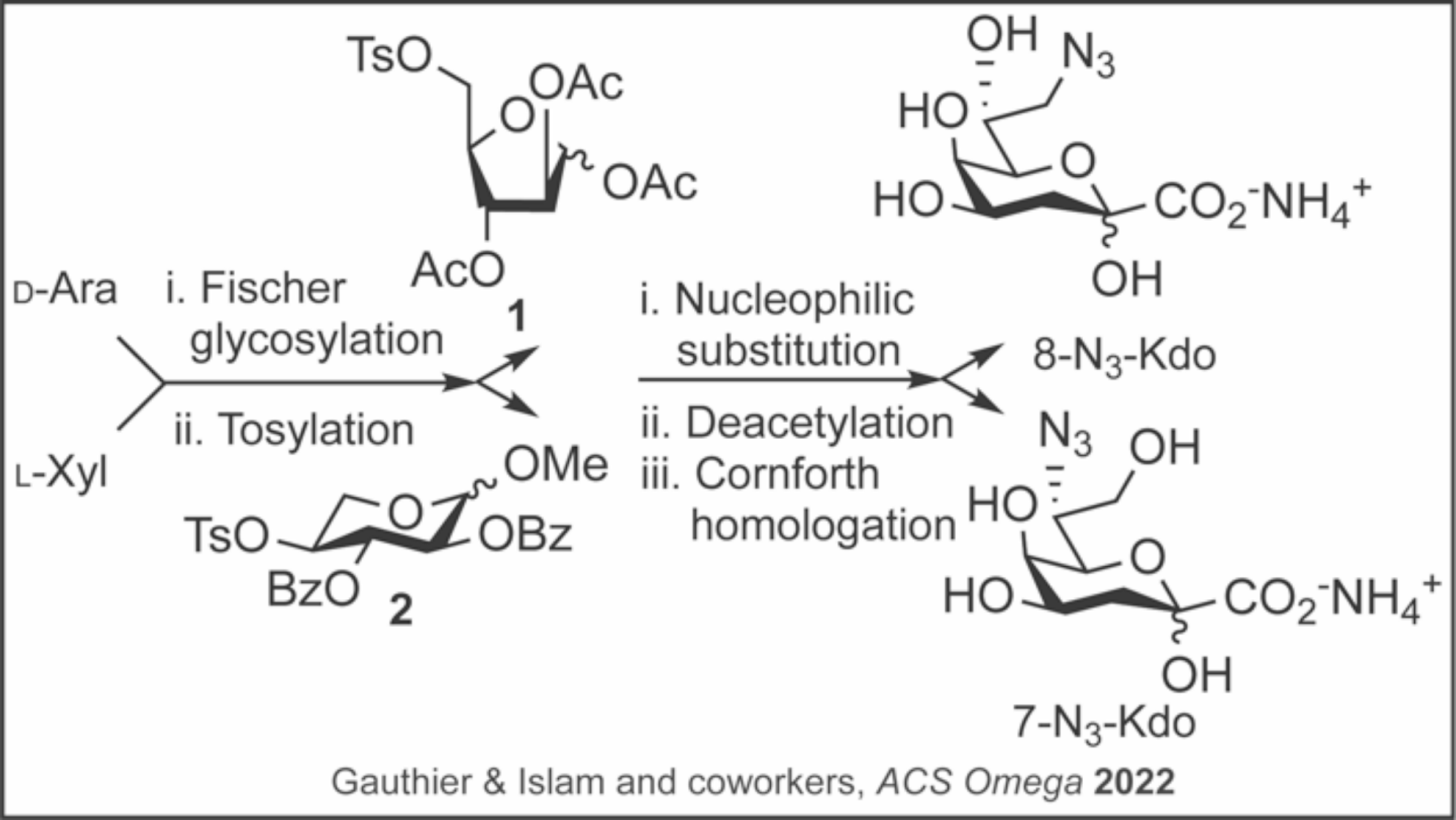
**Synthesis of 8-N**_**3**_ **-Kdo and 7-N**_**3**_ **-Kdo^a^**

a Abbreviations: Ac, acetyl; Bz, benzoyl; Me, methyl; Ts, tosylate.

While Kdo is found across Gram-negative species, more recent work has focused on the analysis of sugar probes that mimic sugar residues found on the cell surface of particular bacterial strains, which has the potential for the narrow-spectrum detection of target bacteria. The Dube and Kulkarni groups, along with others, have undertaken the design and analysis of various rare monosaccharide mimics carrying bioorthogonal handles to study the incorporation of these probes, such as analogues of the rare sugar bacillosamine and the common sugar GalNAc, into different bacterial species.^22,35^ These previous efforts have been well-reviewed in the last several years elsewhere.^36,37^ Recently, Dube, Kulkarni, and co-workers reported the synthesis and use of azido analogues of rare deoxy amino l -monosaccharides *N*-azidoacetyl-l-fucosamine (l-FucNAz), *N*-azidoacetyl-l-pneumosamine (l-PneNAz), *N*-azidoacetyl-l-rhamnosamine (l-RhaNAz), and *N*-azidoacetyl-l-quinovosamine (l-QuiNAz) that mimicked sugars found in the surface glycans of different bacteria (Figure 2A, (ii)).^35^ The concise syntheses of l-FucNAz and l-QuinNAz start with commercially available l-Rha, while those of l-PneNAz and l-RhaNAz start with l-Fuc (Scheme 2).^35^ Azido groups were added to each precursor sugar prior to reduction and coupling reactions with azidoacetic acid to create amide derivatives (**3a**–**b**, **4a**–**b**).^35^ Hydrolysis of anomeric protecting groups followed by acetylation resulted in target molecules used for biological experiments without further purification.^35^

**Scheme 2:**
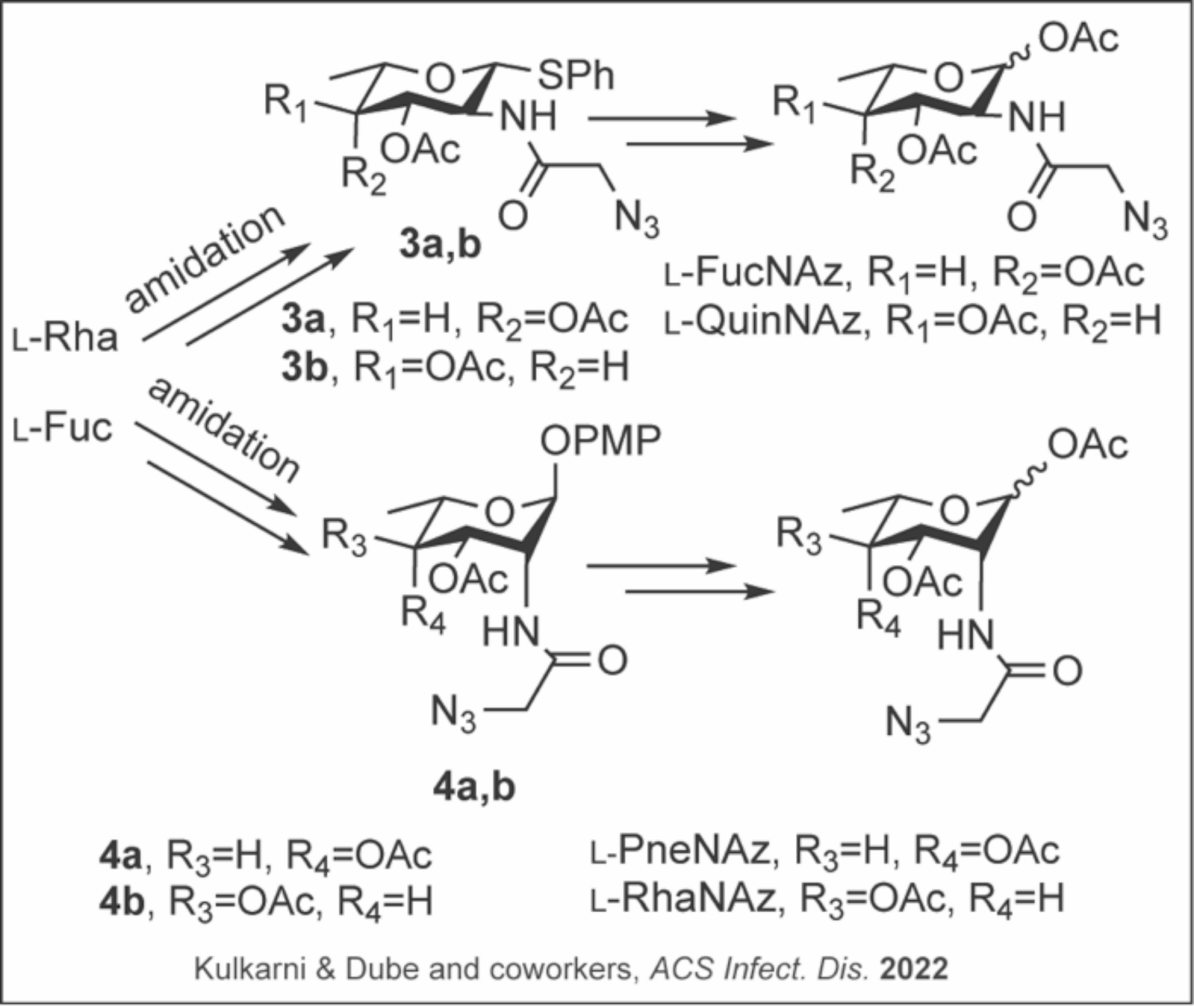
**Synthesis of** ***N*** **-azidoacetyl** l **-Sugars^a^**

a Abbreviations: Ph, phenyl; PMP, *p*-methoxyphenyl.

The native sugars that inspired these azido l-sugars are found across different species. *N*-acetyl-l-fucosamine (l-FucNAc) and *N*-acetyl-l-pneumosamine (l-PneNAc) are present in the LPS of *Plesiomonas shigelloides* serotypes, *N*-acetyl-l-quinovosamine (l-QuiNAc) and *N*-acetyl-l-rhamnosamine (l-RhaNAc) are present in the capsular polysaccharides of *Vibrio vulnificus* strains, and l-FucNAc is also found in the capsular polysaccharides of particular *Staphylococcus aureus* serotypes.^35^ Hence, these bacteria were chosen for initial MOE experiments using the synthetic azido l-sugars that were analyzed via Staudinger ligation or SPAAC with reporters. This work revealed that *P. shigelloides* and *V. vulnificus* proteins were labeled when each of the azido l-sugars was added, while *S. aureus* contained no labeled protein (Figure 2A, (ii)).^35^ Interestingly, even control sugar probes that label the biomolecules of various species, such as *N*-azidoacetylglucosamine (Ac_4_GalNAz), did not specifically label any proteins in *S. aureus*.^35^ On the other hand, flow cytometry analysis of azidosugar-treated cells indicated subtle SPAAC-mediated surface labeling with only some of the l-sugar probes used in *V. vulnificus* but not the other tested microbes.^35^ Further evaluation of these probes with strains known to undergo metabolic labeling indicated that the pathogen *Helicobacter pylori* was able to use all of the l-sugar analogues for incorporation into cytoplasmic and/or surface-exposed glycans, but *Campylobacter jejuni* and *Bacteroides fragilis* showed low or no metabolic oligosaccharide incorporation of azido l-sugars by either assay.^35^ Notably, comparison of immunoblot and flow cytometry analyses yields information on the types of epitopes that are labeled using l-sugar probes, as mainly surface-inaccessible glycans appeared to be labeled in these studies.^35^ Further, l-sugar probes did not label glycans in a human gastric adenocarcinoma (AGS) cell line.^35^

While it was expected that azido l-sugars would not integrate into human glycans that do not contain similar l-aminosugar epitopes, it was surprising that some l-sugar analogues were incorporated into*H. pylori* glycoconjugates, as these analogues are not predicted to mimic native monosaccharide precursors found in*H. pylori*. Hence, these initial studies represent a starting point to further assess how and where rare sugar probes are incorporated into different bacterial species.^38,39^ The substrate scopes of bacterial membrane transporters, esterases for acetyl group removal, sugar kinases, nucleotidyltransferases, and glycosyltransferases may be further explored with these l-sugar probes in species such as *V. vulnificus* and *H. pylori*, where l-sugar incorporation into glycans was observed, but analysis of activated intermediates and final glycan structures has not yet been reported. We anticipate that much will be learned about sugar uptake and metabolism using these synthetic probes.

Other recent applications of rare sugar probes have relied on smaller chemical handles for the selective labeling of bacteria in host cells, which aim to successfully leverage the promiscuity of bacterial biosynthetic pathways while avoiding incorporation into mammalian cells. As there are no probes in clinical use for the visualization of bacterial infections, one promising approach is positron emission tomography (PET) imaging using ^18^F-labeled sugar analogues that replace a single hydroxyl group with a fluoro atom. One broadly used metabolic sugar probe is ^18^F-deoxyglucose (^18^F-FDG), an analogue of 2-deoxy-glucose, which is known to show high levels of uptake in sites of infection and inflammation (Figure 2B).^40^ However, ^18^F-FDG uptake is not unique to inflammation caused by bacteria, and high concentrations of 2-deoxy-glucose inhibit cell growth of some Gram-positive species. A 2017 study showed that ^18^F-FDG was taken up by >20 clinical isolates, including *E. coli*, *P. aeruginosa*, *K. pneumoniae*, *S. aureus*, and *Streptococcus pyogenes*, which set the stage for using bacteria-specific sugar analogues as PET tracers.^41^ Notably, the Glc analogues used were not acetylated, and deletion of phosphotransferase genes in *Bacillus subtilis*, which mediate free Glc uptake and phosphorylation, resulted in a decrease in bacterial cell labeling, which provides a mechanism of uptake in these cells.^41^ In 2023, the Swenson group developed a series of ^18^F-labeled l-Rha derivatives based on previous synthetic fluoro sugar scaffolds.^42–44^ Synthesis of radioactive compounds (**8a**–**c**) commenced from commercially available l-Rha, methyl-l-rhamnopyranoside, and l-mannose (l-Man), respectively (Scheme 3).^45^ The syntheses of triflate precursors^44^ (**5–7**) were achieved with minor modifications. After manual optimization of sugar labeling efficiency with the desired F isotope, followed by deprotection, the automated syntheses of ^18^F-labeled l-Rha analogues were carried out using an automated module.^45^

**Scheme 3:**
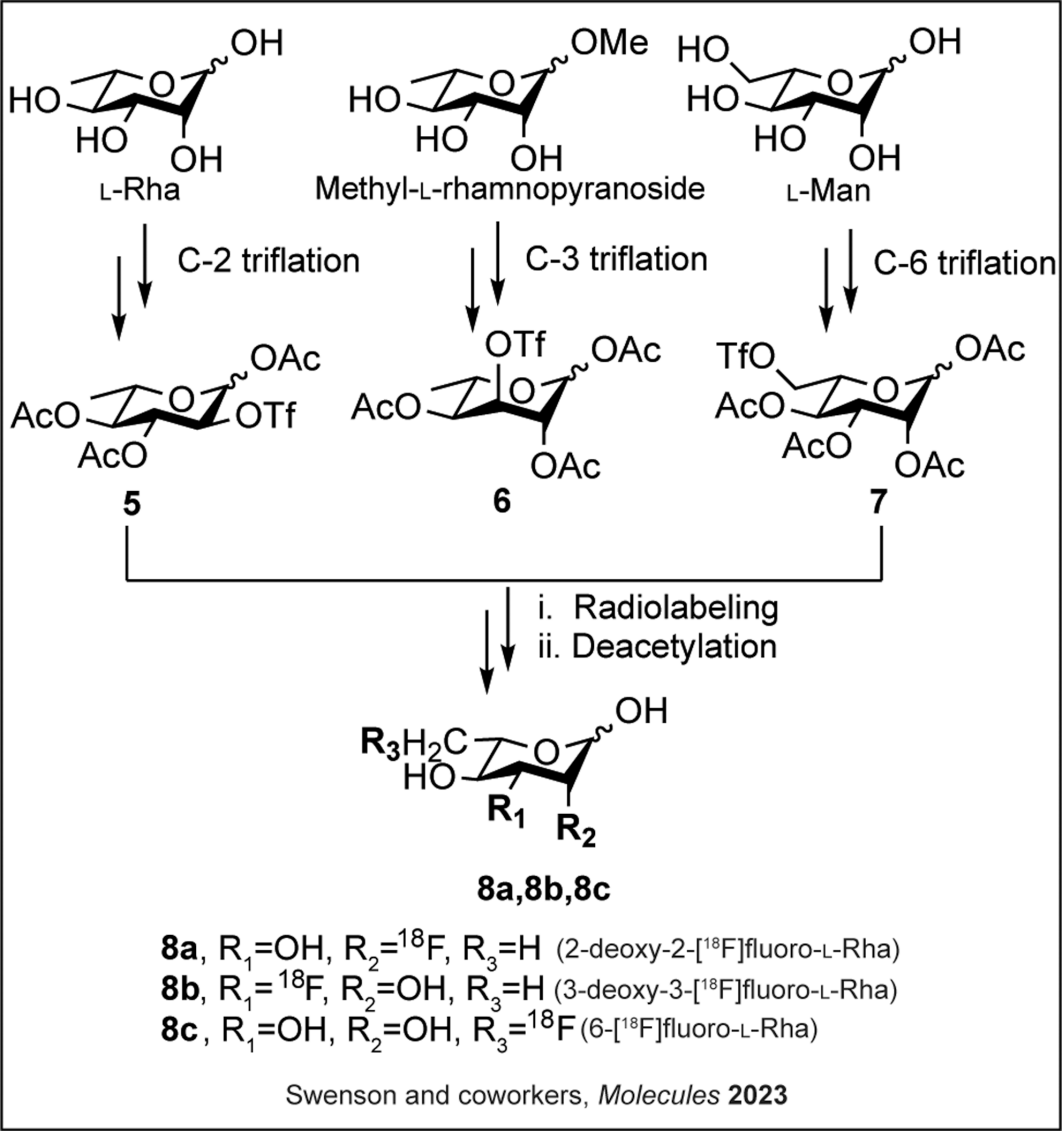
**Synthesis of** ^**18**^ **F-labeled** l **-Rha Derivatives** ^a^

a Abbreviation: Tf, triflate.

Preliminary *in vivo* PET imaging in mice showed that 2-deoxy-2-[^18^F]­fluoro-l-Rha (**8a**) was stable for at least 1 h in animals and several hours in human serum, with no accumulation in major organs and successful renal clearance (Figure 2B).^46^ On the other hand, 3-deoxy-3­[^18^F]­fluoro-l-Rha (**8b**) and 6-[^18^F]­fluoro-l-Rha (**8c**) were metabolized in mice, as they were observed to be rapidly defluorinated, indicating that the position of the fluoro group is important in the selection of an appropriate probe for labeling of bacteria in mammalian hosts.^46^ Notably, previous analysis of 2-deoxy-2-fluoro-l-Rha as an l-Rha inducer analogue in *E. coli* indicated that the analogue induced modest expression of the *rhaBAD* operon, suggesting that it is taken up by the l-Rha:proton symporter RhaT and recognized by l-Rha-binding proteins, which is promising for its use as a probe in bacteria.^44^ Future work involves the analysis of 2-deoxy-2-[^18^F]­fluoro-l-Rha probes in infected animals to observe whether selective accumulation occurs in bacterial cells, which would demonstrate the true orthogonality of l-sugar biosynthetic systems.

Finally, it should be noted here that a recent study by Withers and colleagues uncovered important insights about azide-substituted sugars, as there are obvious differences in size and structure between azide groups and the hydroxyl groups that they often substitute.^43^ The authors systematically analyzed a series of fluorescent Glc/GlcNAc analogues, each carrying an azido group at different positions of the sugar ring, for hydrolysis by hundreds of active glycosidase family enzymes.^43^ Through comparisons of kinetic parameters of each substrate, they found that 6-azido-modified sugar substrates were the best tolerated substrates, while analogues with an azide at secondary carbon atoms were typically not utilized as substrates.^43^ None of the tested GlcNAc analogues were utilized by *N*-acetylglucosaminidases at specificity constants that exceeded more than 10% of that of the native substrate modified with a reporter handle.^43^ As this study was performed with only single enzymes, the authors stress that examples of successful incorporation of unnatural sugar probes into cellular glycans can be accomplished only in biosynthetic pathways that involve many appropriately permissive glycan-processing enzymes. Importantly, many of the recent azido sugar probes are derivatized at amino groups in sugars of interest, which likely bind enzymes that have larger recognition pockets for native substrates that contain –NHAc groups. Alternatively, fluoro sugar analogues, as opposed to azido sugars, have been used to produce inhibitors of sugar-processing enzymes in cells,^47^ and new applications of the described imaging studies will likely continue to shed light on other uses of these probes. Hence, there is much to be learned from the use of sugar analogues carrying chemically diverse functional groups in the biochemistry of rare sugar biosynthetic pathways.

### Enzymatic and Directed Evolution Strategies toward Activated Rare Sugars and Bacterial Glycans

Several barriers still exist toward the synthesis of rare nucleotide sugars that limit our ability to assess the substrate scopes of sugar-processing enzymes *in vitro*, which would provide greater insight into the structural diversity of glycan products that may be produced in cells. This section focuses on several recent reports on enzymatic methods to activate bacterial sugars. Additionally, we highlight glycan metabolism proteins that have been engineered via rational or directed evolution approaches to expand their substrate tolerance to include non-natural sugars. Several impactful campaigns in the directed evolution of bacterial glycan enzymes have been conducted in the past. Notably, the Thorson group has demonstrated the expanded substrate tolerance of the nucleotidyltransferase RmlA for a broader range of non-native sugar-1-phosphate substrates to produce a nucleotide l-sugar, as well as broadened substrate specificities in the donor and acceptor pockets of the natural product glycosyltransferase OleD to produce new biomolecular scaffolds.^48,49^ These efforts provide a strong foundation for ongoing work in this field.

Recent elegant “cascade conversion strategies” utilize fully enzymatic methods to access rare nucleotide sugars on multigram scales. In 2022, Wen and co-workers demonstrated that they could produce 13 different nucleotide sugars, many bacteria-specific, starting from common Man, sucrose, or other sugar building blocks (Figure 3A).^50^ All of the nucleotide sugars were obtained in overall yields exceeding 60% and involved coupled reactions of two–four enzymes. As bacterial glycomes are enriched in dTDP conjugates,^9^ the Wen group then disclosed enzymatic routes to 20 different dTDP sugars in 2023. Their routes began with the key metabolic precursor dTDP-Glc, which was produced from sucrose, resulting in mainly activated d-sugars.^51–54^ Similar to their previous work, this 2023 report takes advantage of cofactor regeneration systems to drive the formation of products with low concentrations of added cofactors and nucleotides for the donation of phosphate groups.^52,55–59^ In addition to optimizing the synthesis of the prevalent activated rare sugar dTDP-β-l-Rha, the authors used enzymes originating from various bacteria and viruses to access several dTDP-activated amino deoxysugars including dTDP-d-FucNAc and dTDP-d-QuiNAc.

Several other groups have focused on enzymatic routes to GDP- and UDP-sugars recently,^60^ many of which are utilized across mammals, plants, and microbes and have been reviewed elsewhere.^61,62^ Notably, the Grimes group has reported detailed protocols for the use of bacterial recycling enzymes to produce the activated bacterial sugar UDP-MurNAc and analogues of the common sugar UDP-GlcNAc that contain biorthogonal handles.^63^ They also performed kinetic analyses to compare the efficiency of routes with different monosaccharide starting materials. Similar quantitative analyses of other enzymes involved in nucleotide rare sugar synthesis would provide useful details about the efficiency of other published coupled reactions. Collectively, these efforts provide new biochemical strategies to synthetically challenging nucleotide sugars that will make downstream studies of glycosylation more accessible for other researchers.

**Figure 3:**
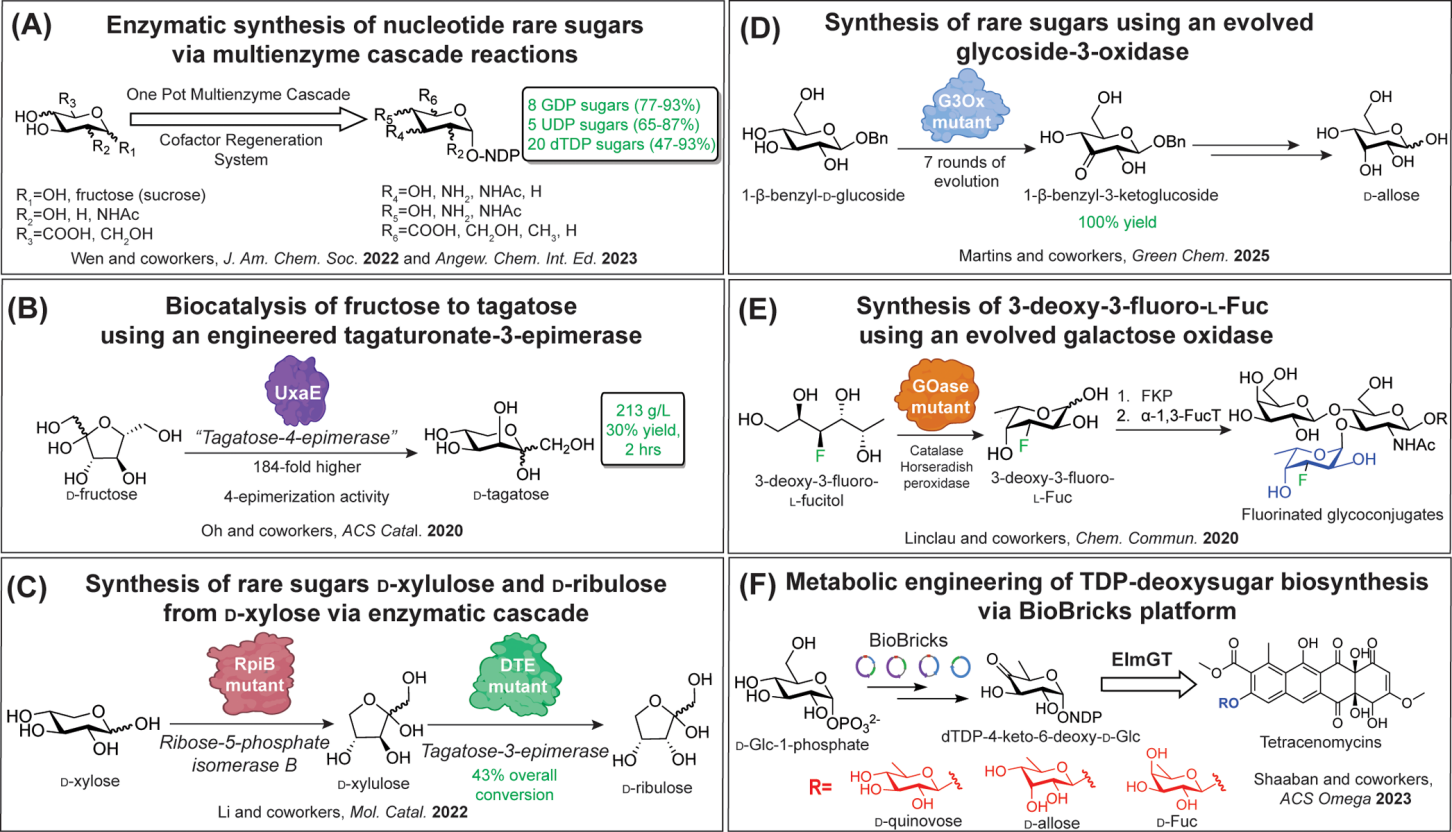
Enzymatic and directed evolution strategies produce activated rare sugars and glycans. (A) Nucleotide sugars synthesized via multienzyme cascade reactions, resulting in high yields of >30 activated sugars, many of which are prokaryote-specific. (B) Engineered tagaturonate-3-epimerase used to produce the rare sugar tagatose from fructose in one step. (C) Multiple engineered enzymes coupled to synthesize rare sugars xylulose and ribulose. (D) Evolved glycoside-3-oxidase used to synthesize the rare sugar allose using enzymatic and chemical methods. (E) Evolved galactose oxidase used to produce fluoro-l-Fuc for the chemoenzymatic synthesis of fluorinated glycoconjugates to use as structural probes (FucT = fucosyltransferase). (F) “BioBricks” platform developed to enable metabolic engineering of d-configured dTDP-deoxysugar substrates of the promiscuous glycosyltransferase ElmGT to produce natural product analogues.

As enzymatic methods continue to be developed for new routes toward glycan precursors, protein engineering strategies have expanded the scope of microbial sugar synthesis in the last several years. Similar to cascade reactions, some of these approaches take advantage of the Izumoring strategy, named after Prof. Izumori, by which rare sugars are produced through cycles of enzymatic epimerization, isomerization, and redox reactions.^64,65^ In 2020, Oh and workers engineered an epimerase enzyme to develop an efficient route to the “rare” ketohexose d-tagatose, which is a low-calorie sweetener found in low quantities in nature and an epimer of d-fructose (Figure 3B). d-tagatose is typically produced from the isomerization of d-Gal derived from lactose; however, simpler one-step syntheses are in demand. A *Thermotoga petrophila* tagaturonate-3-epimerase (UxaE) was reported to catalyze the isomerization of hexuronates d-fructuronate and d-tagaturonate via modification of the C(3) position. In this work, UxaE was engineered to increase its 4-epimerization activity towards hexose d-fructose to produce d-tagatose in one step.^66^ They did so by generating a homology model of *T. petrophila* UxaE, followed by ligand docking to perform a favorable interaction count of critical active site residues.^66^ Based on these analyses, residues were chosen for site-directed and saturation mutagenesis based on their predicted proximity to d-fructose and a lack of interactions with the native substrate d-fructuronate.^66^ Thousands of variants were tested, resulting in a five-site mutant with the desired increase in catalytic efficiency toward d-fructose and decreased efficiency toward the native substrate.^66^ The final tagatose 4-epimerase produced d-tagatose from d-fructose in 30% yield with a productivity of 106 g/L/h.^66^ Similar protein evolution strategies may be used for the improved production of other desired rare sugars for which relevant protein structures do not exist, as homology models may be built as a starting point for future directed evolution efforts of similar enzymes.

Other engineered enzymes have been incorporated into cascade approaches to generate multiple rare sugar products. A recent example was provided by Ju, Li, and co-workers in 2022, as they showed that ribose-5-phosphate isomerase B could be engineered to optimize the conversion of the abundant common monosaccharide d-xylose to the rare sugar d-xylulose. *Pseudomonas cichorii* d-tagatose 3-epimerase, which was already shown to be promiscuous,^67^ was then added to d-xylulose to produce another valuable rare sugar, d-ribulose (Figure 3C).^68^

Alternative approaches have also emerged through the engineering and evolution of commercially relevant proteins. A recent example was published in this year, in which Martins and co-workers developed an improved one-step strategy toward the rare noncaloric sweetener and commercially used rare sugar d-allose from its C(3)-epimer Glc (Figure 3D).^69^ The authors engineered a bacterial glycoside-3-oxidase (G3Ox) that typically modifies C-glycosides at the C3 position to optimize its residual activity for d-Glc.^69^ G3Ox underwent error-prone PCR- and DNA shuffling-based evolution to produce ∼50 K variants that were screened for enhanced catalytic activity with d-Glc.^69^ Interestingly, the final hit variant showed a >20-fold increase in the *k*_cat_ for d-Glc compared to the wild-type enzyme, but a similar *K*_M_ value.^69^ However, the *K*_M_ decreased by 25-fold when an aromatic protecting group was added to the anomeric position of d-Glc to produce 1-*O*-benzyl-β-Glc, which was hypothesized to participate in more noncovalent interactions than Glc with the enzyme’s active site.^69^ Regioselective C(3) oxidation of 1-*O*-benzyl-β-glucose was followed by stereoselective chemical reduction of the C(3) position, followed by deprotection of the anomeric position to produce d-allose in 81% overall yield.^69^ As the evolved G3Ox enzyme shows activity toward other monosaccharides, this method can be applied to the production of other C(3) epimers, such as the rare sugars d-gulose and d-altrose, using d-Gal and d-Man as starting materials, respectively. Importantly, this work highlights the potential of combining directed evolution and organic synthesis to produce desired sugar products.

Additionally, enzymatic strategies have been developed for the synthesis of activated l-deoxysugar analogues for incorporation into glycans to produce probes or inhibitors. One recent example was reported by Flitsch, Turnbull, Linclau, and co-workers on the chemoenzymatic synthesis of modified glycans using activated 3-deoxy-3-fluoro-l-Fuc (Figure 3E).^70^ The authors utilized previously evolved variants of Gal oxidase, which were shown to have activity against a variety of primary and secondary alcohols, to oxidize 3-deoxy-3-fluoro-l-fucitol to 3-deoxy-3-fluoro-l-Fuc in a one-pot reaction with coupled catalase and peroxidase activities.^70–72^ A bifunctional fucokinase/l-Fuc-1-phosphate-guanylyltransferase (FKP), which is known to utilize a variety of l-Fuc analogues as substrates, was then used to activate this analogue to the corresponding GDP-3-deoxy-3-fluoro-l-Fuc so that it could be used as a glycosyl donor.^73,74^ While 2-fluoro-l-Fuc is known to inhibit fucosylation, the activated 3-fluoro-l-Fuc could be utilized by an *H. pylori* α-1,3-fucosyltransferase to synthesize a fluorinated Lewis x analogue carrying an azide handle. Similarly, an azide-labeled type I H-antigen could be prepared using another fucosyltransferase, showing the versatility of the approach. We envision that this strategy could be applied to fluorinated analogues of other rare sugars, such as the l-Fuc analogue l-colitose (l-Col), to produce modified glycans to use as probes to study interactions of uniquely labeled glycans with receptors using NMR spectroscopy analysis, as performed with other fluorinated sugars.^75^

Combinatorial biosynthetic approaches have also emerged over the last several decades that take advantage of biological hosts and genetic engineering strategies to append noncanonical sugars to natural products by the expression of both promiscuous sugar-activating enzymes and glycosyltransferases without the need for protein purification. As many natural products are derivatized with microbial rare sugars, the literature is rich with examples of mixed natural product gene cluster expression and “glycorandomization” of chemical scaffolds of interest, which has been reviewed extensively elsewhere.^61,76^ One rare sugar-derivatized natural product that has long been the subject of metabolic engineering efforts is elloramycin, an aromatic polyketide that contains a permethylated 8-*O*-l-Rha, produced in *Streptomyces olivaceus*. Elloramycin shows antimicrobial activity against Gram-positive species and anticancer properties via ribosomal translation inhibition in human cells; however, the role of l-Rha in the latter activity is not well understood. ElmGT is the glycosyltransferase that attaches l-Rha to the elloramycin aglycone (8-demethyl-tetracenomycin C) and exhibits a broadened donor scope for different dTDP-l-/d-deoxysugars.^77–80^ In 2023, the Nybo, Metsä-Ketelä, and Shaaban groups reported a method to streamline the production of defined glycosyl donors for ElmGT using a synthetic biology “BioBricks” platform, in which TDP-deoxysugar biosynthetic operons were cloned into separate sugar plasmids that are coexpressed in a recombinant *Streptomyces coelicolor* strain with optimized production of ElmGT and 8-demethyl-tetracenomycin C acceptor scaffold (Figure 3F).^81,82^ As many activated deoxysugars, including dTDP-l-Rha, are derived from the common precursor dTDP-4-keto-6-deoxy-d-Glc, the authors first investigated the compatibility of “mixed and matched” dTDP-l-Rha biosynthetic genes from several different antibiotic biosynthetic gene clusters and identified optimal combinations for the production of elloramycin.^81^ Selected gene cassettes were then used to produce other dTDP-deoxysugars as donors, resulting in new elloramycin analogues, including those functionalized with d-Fuc, d-allose and d-quinovose.^81^ While resulting analogues lacked antiproliferative activity against several tested human cancer cell lines, some showed antibacterial promise, highlighting the importance of the sugar moiety in the bioactivity of these compounds.^81^

There remain many applications of enzymatic approaches toward nucleotide sugars and glycans that can be exploited in the coming years. Notably, the expansion of rare sugar glycosyltransferase characterization beyond those involved in the derivation of well-studied natural products and prevalent cell envelope precursors will provide more efficient routes to rare sugar-containing glycans. For instance, our laboratory has recently reported the characterization of the rhamnosyltransferase*E. coli* WbbL for the production of various microbial glycolipid analogues,^83^ and the Imperiali and Allen groups have demonstrated the diversity of activated common and rare sugar substrates for glycosyltransferases from *C. jejuni*.^84^ Further, “BioBrick” approaches toward the modular biosynthesis of glycans containing rare sugars will likely be utilized to study the structure–activity relationships of sugar moieties on various scaffolds.^81^ New approaches that exploit click chemistry with released azide-functionalized glycan substrates,^85^ as well as fluorescently labeled acceptor substrates, for the evolution of glycosyltransferases^86^ offer much promise for new and flexible routes toward glycans containing diverse rare sugar structures.

### Rare Sugar-Mediated Recruitment of Carbohydrate-Binding Protein (CBP) Partners and Their Biological Applications

The complementary methods of glycan microarray-based analyses,^87^ as well as antibody and lectin microarrays,^88,89^ have shed much insight into the diversity of glycan sequences expressed on bacterial surfaces and their interactions with CBPs, namely, antibodies and lectins.^90^ The use of miniaturized microarray formats for the study of bacteria–host interactions has been well-reviewed by others in the last several years.^87,88,90–92^ Pioneering studies on the construction of microbial glycan arrays obtained from natural sources and enriched in bacterial sugars were conducted by Wang and co-workers over 20 years ago.^93^ Later, Paulson, Cummings, and co-workers used purified bacterial polysaccharides to build routinely used microbial glycan arrays containing >300 antigens.^94^ While these glycan microarrays provide a powerful tool for the discovery of CBPs for microbial detection, it remains challenging to validate key sugar motifs involved in glycan–CBP interactions using these complex samples. Accordingly, the use of well-defined glycan motifs arrayed by the Gildersleeve laboratory and others has demonstrated that random human serum samples contain high levels of antibodies against l-Rha conjugates compared to other analyzed glycan motifs.^95,96^ Because of their absence in humans and high solubility, l-Rha and other nonhuman sugars, such as Gal-α1,3-Gal (αGal), have been widely exploited as antibody-recruiting molecules (ARMs) for various applications, namely, cancer cell targeting.^97^ In this section, we focus on the development of rare sugar-containing probes, many of them containing l-Rha, for capturing CBPs to provide greater insight into the modes by which rare sugars mediate host–pathogen interactions and the applications of these glycan–CBP interactions. Notably, since many bacterial surface glycans are immunogenic and have been synthesized or purified to be used as antigens for vaccine development,^19^ there are several other applications that fall out of the scope of this perspective.

ARMs serve as a bridge between target cells and endogenous antibodies found in a given host. They are generally composed of the two binding motifs: the tumor-binding molecule (TBM) and the antibody-binding molecule (ABM).^97^ While TBMs are often antibodies or peptides that can bind to the various parts of tumor cells, ABMs are usually small haptens, including l-Rha, αGal, and 2,4-dinitrophenol (DNP), which bind to endogenous antibodies abundant in human serum (Figure 4A).^97^ l-Rha is the most utilized hapten, as human sera are known to have higher anti-Rha than anti-αGal or anti-DNP.^96^ Accordingly, there is some initial work by Bernardi and co-workers to develop l-Rha-based glycomimetics containing a thioether linkage, proposed to be more hydrolytically stable than native glycosidic bonds, for in vivo applications (Figure 4B).^98^

**Figure 4:**
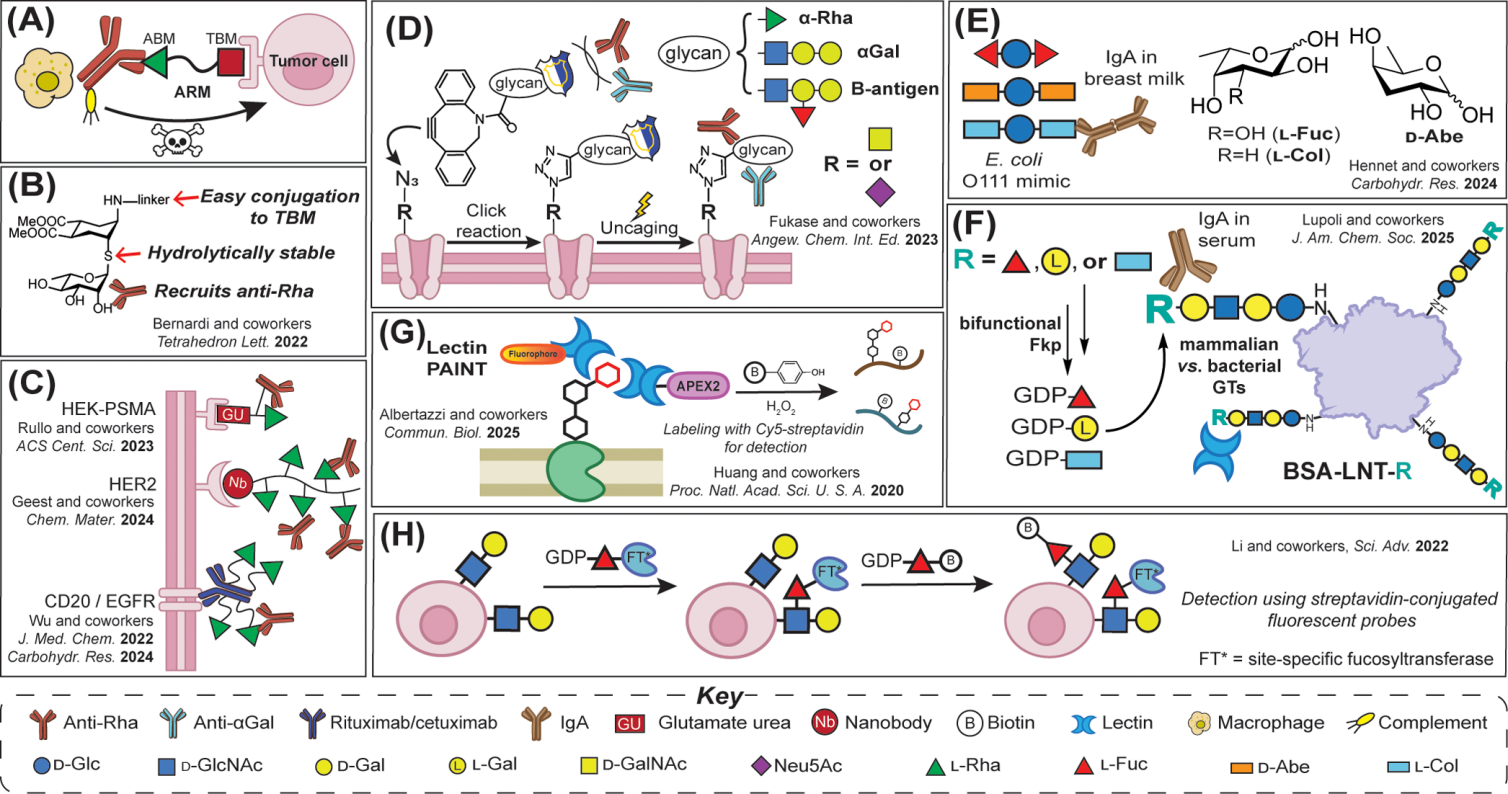
Protein-based recognition of rare glycan motifs and applications for carbohydrate-binding protein (CBP) recruitment. (A) Antibody-binding molecule (ARM) used for the recruitment of CBPs to mediate complement-mediated cytotoxicity against tumor cells (ABM is the antibody-binding molecule; TBM is the tumor-binding molecule). (B) Rha-based glycomimetic synthesized to improve the hydrolytic stability of ARMs. (C) cARMs produced to covalently engage anti-Rha antibodies to overcome the inherent low affinity of antibody–monosaccharide interactions (top). Multivalent presentation of Rha polymers used to enhance recruitment of anti-Rha antibodies to tumor cells (middle, bottom). (D) Glycan-caging strategy coupled with metabolic oligosaccharide incorporation of azido sugars in tumor cell surfaces, leading to a method that prevents the premature engagement of glycan antigens by CBPs, resulting in improved cytotoxicity of target cancer cells. (E) High levels of IgA antibodies against synthetic *E. coli* O111 trisaccharide mimics with terminal l-Col detected in human breast milk samples. (F) Chemoenzymatic incorporation of terminal l-sugars on BSA-lacto-*N*-tetraose (LNT) conjugates showing high levels of IgA antibodies with specificity for l-Col and not other related l-sugars in random human serum sample. (G) Proximity labeling accomplished with lectin-APEX2 conjugates to identify new CBPs and glycoprotein-binding partners (*right*). Lectin-PAINT was developed for the detection of lectin-binding sugars using super-resolution microscopy (*left*). (H) FucID method leveraging a promiscuous fucosyltransferase and GDP-l-Fuc analogues for intracellular proximity labeling of target molecules on cell surfaces.

To improve the low affinities inherent in monosaccharide–antibody interactions (*K*_D_ ∼ 0.001–1 mM), Rullo and co-workers recently used their developed covalent ARM (cARM) strategy to covalently engage recruited anti-Rha antibodies with target cells (Figure 4C, *top*).^99^ cARMs were equipped with a monomeric l-Rha as the ABM, a reactive electrophile for covalent engagement of recruited anti-l-Rha antibodies, and a glutamate urea (GU) moiety as a TBM that interacts with prostate-specific membrane antigen (PSMA) on cancer cells for “tumor-immune proximity induction” to clear tumors.^99^ The authors compared the behavior of cARMS containing acyl imidazole versus sulfonyl chlorides capable of sulfur fluoride exchange (SuFEx) chemistry and found that the latter label monoclonal antibodies ∼50-fold faster than the former.^99^ Treatment of PSMA-expressing cancer cells with cARMs containing SuFEx handles showed target opsonization with anti-l-Rha antibodies, which could be visualized by microscopy; comparable opsonization was not observed at similar concentrations of ARMs lacking covalent handles.^99^ Overall, this work demonstrates the usefulness of covalent handles in compensating for weak l-Rha–CBP interactions to promote approaches for the antibody labeling of target cells.

Notably, the approach by the Rullo group avoids multivalent l-Rha presentation, which can cause nonselective “target agnostic activation”. Recent work by Geest and co-workers aimed to improve the selectivity of multivalent displays toward target tumor cells by fusing multivalent l-Rha polymers to nanobodies that directly bind to antigens presented on cancer cells (Figure 4C, *middle*).^100^ Similarly, over the last several years, Wu and co-workers sought to enhance the effector function of clinically used monoclonal antibodies for cancer immunotherapy by conjugating multiple l-Rha moieties directly on rituximab (RTX), a clinically approved anti-CD20 monoclonal antibody, and cetuximab (CET), a human/mouse chimeric anti-EGFR monoclonal antibody (Figure 4C, *bottom*).^101,102^ Both antibody–l-Rha conjugates caused an increased recruitment of endogenous anti-Rha antibodies to target tumor cells and enhanced complement-dependent cytotoxicity clearance pathways. Taken together, these reports highlight the many applications of l-Rha conjugates for recognition by CBPs for immune activation, which has been supported by the work of other groups.^103^

Although endogenous antibody recruitment with l-Rha-based chimeras has advanced over the last several years, one lingering challenge is that the hapten molecule can be sequestered by endogenous antibodies prior to target cell binding. To promote initial target engagement, Fukase and co-workers developed an elegant de novo glycan display approach that combines the metabolic labeling of tumor cells and a glycan-caging strategy to delay the capture of l-Rha by endogenous antibodies until incorporation of the antigen on the cell surface has occurred (Figure 4D).^104^ Tetraacetylated azido sugars, *N*-azidoacetylmannosamine (ManNAz) or *N*-azidoacetylgalactosamine (GalNAz), were metabolically incorporated into the surface of B-cell lymphoma cells, which were then click-conjugated with the dibenzocyclooctyne (DBCO)-functionalized B-antigen, αGal or l-Rha.^104^ Cells labeled with B-antigen or αGal showed comparatively less complement-mediated cytotoxicity than l-Rha-labeled cells, again likely because humans often contain more anti-Rha than other antiglycan antibodies.^96^ A photocleavable protecting group was appended to the C(3) position of the l-Rha bioorthogonal probe, which prevents the binding of anti-Rha antibodies until uncaging via UV irradiation.^104^ They demonstrated that uncaging of l-Rha post-incorporation led to improved killing of cancer cells compared to the unprotected control in the presence of human serum.^104^ This strategy demonstrates that temporally controlled immune responses to tumor cells are possible using caged l-Rha probes. We anticipate that other caged rare sugar probes could be used for studies that aim to detect and engage rare sugar CBPs in complex environments.

Clearly, much of the previous work has focused on the development of rare sugar probes containing l-Rha to exploit known host protein interactions with foreign sugars. However, hundreds of microbial rare sugars exist for which defined probes are unavailable. Recently, several groups have produced defined glycan probes containing rare sugar motifs beyond l-Rha to better detect and characterize microbial sugar–CBP interactions. In 2024, Hennet and co-workers chemically synthesized mimics of the terminus of the O-unit from *E. coli* O111,^105^ which contains d-Glc linked to two rare dideoxy l-Col motifs that are believed to be antigenic (Figure 4E).^106–108^ To assess the specificity of CBP interactions with these motifs, trisaccharide analogues were also synthesized that contained the dideoxy rare sugar d-abequose (d-Abe) or the common deoxysugar l-Fuc as the two terminal residues. Microarray-based analysis of these trisaccharides versus purified LPS containing deoxysugars revealed that human breast milk samples are enriched in IgA antibodies that selectively react with l-Col and showed little to no cross-reactivity with the other tested probes. Importantly, this concentration-dependent interaction between the antigenic sugar and IgA antibodies in complex human samples was difficult to deconvolute using purified O111 LPS, highlighting the value of synthetic probes in the detection of these specific rare sugar–antibody interactions.

Similarly, our laboratory showed in early 2025 that sequence-defined glycoprotein probes that mimic terminal *E. coli* O55 O-Ag or human type 1 H-antigen sequences can be used for rare sugar–antibody detection.^109^ Kim et al. developed concise chemoenzymatic routes using the aforementioned enzyme FKP to produce the activated form of l-Col, GDP-β-l-Col, along with select structural analogues (Figure 4F).^109^ To construct glycan probes containing the l-sugars of interest, the donor substrate scope of the bacterial O-Ag glycosyltransferase WbgN, which is annotated to transfer l-Col, was compared to the human blood group fucosyltransferase FUT2.^109,110^ Both glycosyltransferases showed similar substrate usage and could transfer GDP-activated l-Col, l-Fuc, or the plant rare hexose l-Gal onto free disaccharides or modified BSA glycoproteins that contained a terminal lacto-*N*-biose sequence, which mimics the native acceptor substrate.^109^ The resulting l-sugar-modified BSA probes were then used to survey both commercial and clinical human serum samples, which revealed that some human serum samples contain selective anti-l-Col IgA antibodies.^109^ Additionally, anti-l-Col IgG and IgM antibodies were more abundant than anti-l-Fuc or anti-l-Gal antibodies in most of the examined samples.^109^ Our observations and those of Hennet and co-workers are in accordance with the role of serum and mucosal IgA in binding to microbes as part of host protection mechanisms.^111,112^ As the levels of anti-l-Col and anti-l-Rha vary among individuals, these findings suggest that more rare sugar-binding antibodies might exist in human serum, which could indicate exposure to different pathogens. Further, there is much potential for well-defined glycan probes to be utilized to characterize binding constants and other biophysical parameters of CBP–rare glycan interactions that are difficult to assess with more complex and heterogeneous antigen structures.

In addition to antibodies, lectins are another class of CBPs that are ubiquitous and often serve as an initial defense mechanism against pathogens.^37,92^ While many Rha-binding lectins (RBLs) have been excellently reviewed elsewhere,^91,92^ recent studies have hinted that RBLs are highly overexpressed on cancer cells,^113^ which has led to the development of additional l-Rha probes to mediate labeling of cancer cells and the enzymatic rhamnosylation of anticancer drugs for targeted cancer cell delivery.^114^ The l-Rha moiety not only serves to deliver cargo to RBL overexpressing cancer cells and mediate subsequent internalization into these cancer cells but also limits off-target effects, which provides evidence that rare sugar modification may serve as a promising approach for lectin-based cancer-targeting diagnosis and therapy. Additionally, other rare sugar-binding lectins beyond RBLs have been characterized from invertebrate animals, namely, horseshoe crabs, that do not possess adaptive immune systems and produce many lectins as part of innate immune responses for pathogen recognition.^115^ Most of the characterized horseshoe crab lectins are known to bind LPS, and work in this field has been reviewed recently.^116^ Notably, a series of lectins purified from Taiwanese *Tachypleus tridentatus*, known as tachylectins (TL), bind different sugars or motifs found in bacterial surface glycans, including LPS containing different rare deoxysugars.^117,118^ Since most of this work was conducted over 20 years ago, we anticipate that continued characterization of invertebrate lectin specificities will unveil new tools to study rare sugar recognition.

Beyond the use of native CBPs for rare sugar detection and identification, other modern chemical biology approaches may be extended for the identification of rare sugars on cellular surfaces. In 2025, the Albertazzi group took advantage of the promiscuity of known lectins to develop a technique called “lectin-point accumulation in nanoscale topography” or “lectin-PAINT” (Figure 4G).^119^ The method utilizes lectins conjugated to fluorophores and tracks transient interactions of probes that localize on the cell surface by microscopy to provide multiplex imaging of glycosylation marks at super-resolution of single cells.^119^ The eight plant-derived lectin conjugates were known to detect commonly expressed mammalian glycan motifs containing sialic acid, l-Fuc, Man, Gal, and GlcNAc residues in order to characterize the glycotypes of different cell lines, including cancer-derived cells.^119^ The modification of rare sugar-containing lectins could lead to the expansion of PAINT for the labeling of microbial glycans.

Proximity labeling tools that take advantage of the promiscuity of a single enzyme to label desired glycan motifs on cell surfaces have also been developed. In 2020, the Huang group utilized conjugates of galectins, which are lectins that bind to β-galactosides, for proximity-based labeling of target glycoprotein partners on live cell surfaces (Figure 4G).^120^ Using an engineered ascorbate peroxidase (APEX2) fused to galectin-3, new binders of galectin-3 were identified through covalent modification with a transiently reactive biotin probe, followed by enrichment and mass spectrometry-based proteomic analysis of attached targets.^120^ Further, the Li and Wu groups recently reported an updated version of their “FucID” approach, which facilitates intercellular proximity-based identification of cell–cell interactions using a surface-exposed promiscuous fucosyltransferase (FT) that attaches various labeled GDP-Fuc analogues to nearby “prey” cells containing the disaccharide *N*-acetyl-d-lactosamine (LacNAc) acceptor (Figure 4H).^121^ The FT can attach itself to prey cells by using GDP-Fuc–FT conjugates, followed by subsequent attachment of biotinylated GDP-Fuc for several downstream applications.^121^

Similar approaches to those developed for eukaryotic glycans may be modified for the detection of bacterial surface glycan sequences through further discovery or characterization of rare sugar CBPs. These discoveries may lead to the identification of reporter sugars on cell surfaces for targeting other disease-related cells. Further, rare sugar glycosyltransferases that exhibit permissive donor scopes may be used for the attachment of rare sugars to cell surfaces.^3,83,84^ There is also much potential for biochemists to analyze the binding parameters of rare sugar–CBP interactions to better understand how common and rare sugars with minor structural differences can be distinguished by host immune cells.

### Opportunities for Glycan Sequencing and Computational Tools for Rare Sugar Glycan Analysis

Compared to the structures of nucleic acids and proteins, glycans exhibit much more structural variation due to the presence of different isomers of given sugars (d- vs l-), varying sugar ring sizes and number of carbon atoms, and functional group modifications (such as *N*-acetyl, deoxy positions, etc.).^122^ Additionally, glycans can be branched with alternative glycosidic linkages, which adds to their vast structural diversity. Further, glycans are not directly genetically encoded, so analysis of sugar residue identities cannot be carried out using DNA-based amplification and sequencing methods. While sequencing approaches are laborious for all glycans due to the diversity of rare sugar structures, microbial glycan sequence determination remains an even greater challenge. For instance, the characterization of bacterial O-Ag sequences alone has been carried out by careful analysis of purified samples by two-dimensional ^1^H and ^13^C NMR spectroscopy techniques, in addition to other analytical techniques that have been recently reviewed elsewhere.^13,122,123^ Hence, more qualitative methods such as lectin arrays are often coupled with more quantitative approaches such as mass spectrometry, along with computational analysis, to enable faster delineation of glycan sequences. In this section, we highlight recently developed glycan sequencing technologies that have already been used for the identification of rare sugars within glycans or have the potential to be used in future applications.

Recent work has sought to provide higher-throughput pipelines for lectin-array-type analyses to improve our toolbox for rapid glycan identification. The Kiessling Lab has recently developed a new lectin-based glycan sequencing method called “Lectin-Seq”, in which human lectins were labeled with fluorescent antibodies and incubated with complex microbial samples, followed by fluorescence-activated cell sorting (FACS) for isolation of bound microbes that are subsequently identified by metagenomic sequence analysis (Table 1).^89^ Downstream analysis of lectins with bound bacteria provided information on microbial surface sugars that serve as ligands.^89^ While the focus of this report was on the characterization of soluble human lectins and their microbial partners within gut microbiome samples, the use of lectins displaying epitope tags for detection by fluorescence antibodies that recognize these epitopes makes Lectin-Seq amenable to the use of new lectins for the detection of their unique binding partners.

**Table 1:** Currently Used Experimental and Computational Glycan Sequencing Techniques

**technique**	**relevant application for technique/tool**	**computational tool**	**relevant reference(s)**
^**1**^ **H NMR/** ^**13**^ **C NMR**	commonly used technique for determining glycan structures; a tool recently used for chemical shift prediction of O antigen structure	Geqshift	(Rönnols and co-workers **2024)**
**LC–MS/MS** ^a^	commonly used technique for determining glycan sequences; a tool recently used for the prediction of *N*- and *O*-linked glycoprotein and glycosphingolipid structures	CandyCrunch	(Bojar and co-workers, **2024**)
**MALDI-TOF** ^a^	recently used for the analysis of polysaccharides, including for single-colony bacterial serotyping based on the sequence of expressed O antigens		(Hinou and co-workers, **2022**)
**MS/MS-IR-CID** ^a^	recently developed for the determination of ring size and anomeric configuration of monosaccharides in rare sugar-containing glycan polymers		(Compagnon and co-workers, **2023**)
**lectin array**	well-established technique involving the immobilization of lectins in an array format to assess ligand specificities for glycan analytes; a tool recently used for lectin ligand specificity annotation		(Mahal and co-workers, **2022);**
		LectinOracle	(Bojar and co-workers, **2022**)
**lectin-seq**	recently developed technique for labeling of microbes with fluorescently tagged lectins, followed by metagenomic sequence analysis of microbes to annotate lectin-binding partners		(Kiessling and co-workers, **2023**)
**glycan array**	well-established technique involving the immobilization of synthetic or purified glycans, or isolated bacteria, in an array format for surveying glycan recognition; a tool used for glycan structural prediction/analysis		(Gildersleeve and co-workers, **2024**)
		SweetNet	(Bojar and co-workers, **2021a**)^b^
		Glycowork	(Bojar and co-workers, **2021b**)^c^
**liquid glycan or lectin arrays (LiGAs, LiLAs)**	library of glycans (LiGAs) or lectins (LiLAs) displayed on phage surfaces to study glycan–protein interactions		(Derda and co-workers, **2023**); (Derda and coworkers, **2024**)
**nanopore analysis**	recently developed glycan sequencing method that couples glycosidase treatment of sugar polymers with analysis of analytes using nanopore sensing; a tool used for initial automated sequence determination	MD simulation	(Gao and co-workers, **2025)**

a Acronyms: LC–MS/MS, liquid chromatography with tandem mass spectrometry; MALDI-TOF, matrix-assisted laser desorption ionization-time of flight; MS/MS-IR-CID, tandem mass spectrometry with infrared radiation and collision-induced dissociation.

b Burkholz, R., Quackenbush, J. and Bojar, D. (2021) *Cell Rep.*, *35* (11), 109251.

c Thomès, L., Burkholz, R. and Bojar, D. (2021) *Glycobiology*, *31* (10), 1240.

Others have utilized data sets from decades of published lectin array experiments for computational models to expand our understanding of native CBP ligand specificities. In 2021, a deep learning algorithm called LectinOracle was reported by Bojar and co-workers, which utilized the sequences of glycans and proteins to predict the ligand specificities of lectins, many of which were shown to agree with known lectin specificities using >500K reported protein–glycan interactions for model training (Table 1).^124^ The authors used their developed SweetNet model, a graph convolutional neural network method, that captures structural motifs such as branching and subunit connectivity in its representation of glycans^125^ and combined it with protein representations that account for parts of proteins learned to be relevant. The resulting LectinOracle model was able to reveal nuanced binding preferences of grouped lectins not evident from sequence similarity alone.^124^ Mahal and co-workers partnered with Bojar to expand upon LectinOracle by using a combination of machine learning and manual annotation to systematically define the binding specificities of 57 commercially available lectins based on data obtained from a previous analysis conducted by the Mahal laboratory of 116 lectins with version 5 of the Consortium for Functional Glycomics glycan microarray (Table 1).^37^ This approach yielded distinct binding preferences for each lectin, which were then grouped into one of the following eight categories based on their glycan motif-binding preferences: (1) Man, (2) complex *N*-glycans, (3) core *O*-glycans, (4) l-Fuc, (5) sialic acid and sulfate, (6) terminal GlcNAc and chitin, (7) terminal Gal and LacNAc, and (8) terminal GalNAc.^37^ Notably, additional ligand preferences of each lectin are listed as “prefers or strongly prefers”, along with motifs that have no impact (“tolerates”) or those that prevent binding (“inhibited by”), the latter of which provides information lacking in most lectin specificity analyses.^37^ Overall, this report provides a useful handbook for the binding specificities of commercially available lectins and reveals necessary distinctions among key motifs that mediate lectin–glycan interactions. Noticeably, deoxysugar-binding annotations were reported only for l-Fuc, as most chemically defined glycan arrays lack rare sugars; hence, much opportunity exists in this space to collect data on CBP–rare sugar-binding specificities to feed into established computational pipelines.

Recent work has exploited genetically encoded biosynthetic systems to build libraries beyond traditional microarray formats for the analysis of interactions of CBP with glycans. In 2023, Derda and co-workers, in collaboration with many others, reported on the next generation of their clever liquid glycan array (LiGA) technology (Table 1),^126^ in which the multivalent expression of distinct glycan sequences on phage is linked to a DNA barcode within the genome of each virion, enabling the “genetic encoding” of displayed glycan sequences and assessment of interactions in solution phase rather than on glass slides.^127^ LiGA was enhanced beyond the original 70–90 displayed small synthetic glycans by expanding the chemoenzymatic synthesis approach to complex *N*-glycan motifs that could be used to assess interactions in living animals.^127^ Accordingly, Derda, Macauley, Mahal, and others recently developed a complementary technique called “liquid lectin arrays” (LiLAs) in which lectins are displayed in a multivalent manner on the surface of the phage to create libraries of CBPs to rapidly profile glycan analytes.^128^ Methods for constructing these phage libraries appear agnostic to the lectin and glycan sequence added to the phage surface.^128^ Further, as the construction of glycan structures is amenable to modification by glycan-processing enzymes, we anticipate that microbial glycosyltransferases or glycosidases might be used to build sequences containing rare sugars that may be paired with new methods for generating CBP libraries to uncover currently unknown rare sugar–CBP interactions.

Beyond the use of glycan and lectin arrays to determine biomarkers for disease states or pathogen-specific motifs, mass spectrometry analysis has improved in recent years to provide glycan sequencing information with higher accuracy. Notably, the Hinou group extended the capabilities of MALDI-TOF/MS from polypeptide-targeted identification of species to the analysis of glycan patterns using a technique that they coined “MALDI glycotyping”.^129^ This technique was recently utilized as part of a workflow for the de novo sequencing of O-Ag polymers from a single colony of Gram-negative bacteria in a 1 h time frame and was applied to the serotyping of an *Edwardsiella tarda* strain that was not known to produce O-Ag (Table 1).^129^ Furthermore, in 2023, the Compagnon lab reported a mass spectrometry pipeline with the goal of overcoming the challenge of discriminating pyranose and furanose ring sizes, namely, in Gal residues that can be found as furanoses in plant and bacterial polysaccharides.^130^ The authors combined tandem mass spectroscopy and infrared ion spectroscopy (MS/MS-IR) with collision-induced dissociation (CID) conditions to determine the ring sizes and anomeric stereochemistry of Gal-containing glycans (Table 1).^130^ This work followed a decade of advances on the use of ion mobility or IR laser spectroscopy to enable isomer discrimination, as reviewed elsewhere.^130–132^ We envision that this technique will be useful for the rapid determination of other rare sugar-containing sequences and offers alternatives to traditional structural determination methods.

Others have recently reviewed the growing ability of artificial intelligence (AI) tools to help overcome traditional challenges in glycan sequencing, structural elucidation, and functional prediction and annotation.^133^ One of the major bottlenecks in glycomics is the determination of sugar structures and sequences based on collected tandem mass spectrometry (MS/MS) datasets. In the last year, the Bojar group reported a Python-accessible deep learning model called CandyCrunch, which has been used for de novo glycan sequencing based on previously collected LC–MS/MS data, offering accurate predictions without reliance on large databases (Table 1).^134^ CandyCrunch was trained on >500K annotated LC–MS/MS spectra of glycans collected from different experimental conditions and leverages specific knowledge of data interpretation to predict glycan structures from various classes, including *N*-linked, *O*-linked, and glycosphingolipids, with ∼90% accuracy.^134^ The best predictions were found for *O*-glycans and free oligosaccharides, and the model can successfully distinguish isomeric structures through their distinct fragmentation patterns.^134^ Notably, the dataset used for training was composed of mass spectrometry analysis of eukaryotic glycan structures; hence, the extension of these models to include microbial glycan MS/MS datasets or applications to examine microbial glycan analytical data represents future opportunities in the field.^134^

Just as CandyCrunch aims to extend analysis of glycan MS/MS data to nonexperts, other Python packages, namely, Glycowork, significantly enhance the efficiency of large glycan dataset analysis by automating processes such as glycan motif annotation, visualization, and integration within relevant databases.^135^ Machine learning models were trained for the extraction of structural patterns, which facilitates the rapid identification and analysis of key glycan features within reported datasets.^135^ These techniques have already been applied to determine the neighboring sequence of the monosaccharide Rha in known bacterial polysaccharides, which showed that l-Rha, d-rhamnose (d-Rha), and *N*-acetyl-d-rhamnosamine (d-RhaNAc) often appear linked to other Rha residues in nature.^135^ These visualizations help others rapidly illustrate how specific glycan types are distributed across different species or taxa.

Finally, this year saw the application of nanopore technology to glycan sequence analysis, which holds the potential to revolutionize glycan sequencing due to its high sensitivity, speed, and low cost. Nanopore analysis is typically carried out by the translocation of target polymers through pore-forming proteins, which creates signature current signals that facilitate the analysis of sequences, such as those of nucleic acids. While previous work has shown that nanopores could be used for glycan detection and/or general structural analysis (*e.g*., branching, chemical modification, etc.),^136,137^ the Wen, Long, and Gao groups successfully developed an initial pipeline to perform glycan sequencing.^138^ The authors treated target glycans with known exoglycosidases prior to translocation through an engineered nanopore that they previously showed could sense different LacNAc-containing polymers,^139^ which were used as models for developing the glycan sequencing workflow. The resulting current data was then used to develop a machine learning algorithm for automated sequence determination.^138^ However, many challenges still remain in the application of these techniques to naturally occurring glycans. Namely, selective glycosidases with known cleavage specificities need to be added to analytes, which can be complicated for sequences of unknown identity and for rare sugar-containing sequences for which few characterized glycosidases exist. Further, the sequences of homopolymers are difficult to resolve using the current workflow. Nonetheless, there are many prospects for biochemists to uncover appropriate enzymes to aid in the development of more rapid methods for sequencing complex microbial glycans.

In addition to chemical biology-based techniques for the elucidation of rare-sugar-containing glycan structures, more traditional methods continue to be improved and coupled with computation to facilitate higher-accuracy polysaccharide sequence determination. For instance, a recent model called GeqShift utilizes graph neural networks for high-performance prediction of NMR chemical shifts for complex carbohydrates (Table 1),^140^ including those of bacterial O-Ag’s. Improvements in these models are predicted as access to experimental analytical data of glycans grows.^140^ Similarly, computational scientists have noted that the field of glycomics, especially the sequence analysis of glycans derived from plants and microbes, will benefit not only from expanded datasets but also from more consistent nomenclature and greater standardization across glycan structure databases by glycochemists and glycobiologists. Undoubtedly, new biochemical tools have great potential to expand our ability to analyze sequences containing mixtures of common and rare sugars.

## Conclusions and Outlook

In conclusion, the biochemical study of rare sugars is an active subfield of glycobiology and glycochemistry that ultimately aims to understand the various roles of microbe-specific sugars in biology. It should be noted that we could not cover all of the recent advancements in the production of microbial glycan probes, such as those of based on mycobacterial cell envelope structures,^141,142^ as many of these tools have been recently reviewed elsewhere.^36^ As demonstrated by the summarized examples, the applications of this research span many disciplines, as new chemistry is needed to develop concise, affordable, and robust routes to rare sugar precursors that can be used for enzymatic and chemical methods to construct more diverse glycans. Further, because of the large chemical space of rare sugar structures, many naturally occurring microbial putative enzymes that activate and transfer sugars, and degrade resulting glycans, remain to be biochemically characterized. The quantitative analysis of the kinetic and binding parameters of these enzymes for their substates and ligands will provide necessary data on catalytic efficiencies and interaction parameters, respectively, that are currently lacking in the field. This information would offer much insight into the most efficient routes to microbial glycans to act as probes or antigens for applications in biotechnology and therapeutics. New datasets could then be fed into computational models to improve their predictive capabilities. We envision that future work will have the greatest impact on glycan sequencing, which may improve the available tools in glycobiology to rival those routinely used for nucleic acids and proteins. Hence, there is much excitement for the future of biochemical analysis of complex, rare sugar-containing polymers in the next decade.

## Abbreviations

d-Glc: d-glucose
d-Man: d-mannose
d-Xyl: d-xylose
d-Gal: d-galactose
l-Rha: l-rhamnose
NDP: nucleoside diphosphate
UDP: uridine diphosphate
GDP: guanosine diphosphate
CMP: cytidine monophosphate
CTP: cytidine triphosphate
LPS: lipopolysaccharide
O-Ag: O-antigen
O-unit: oligosaccharide unit
Kdo: 3-deoxy-d-*manno*-oct-2-ulosonic acid
GlcNAc: *N*-acetylglucosamine
MurNAc: *N*-acetyl muramic acid
CBP: carbohydrate-binding protein
MOE: metabolic oligosaccharide engineering
CuAAc: Cu­(I)-catalyzed azide–alkyne cycloaddition
SPAAC: strain-promoted azide–alkyne cycloaddition
PET: positron emission tomography
^18^F-FDG: ^18^F-deoxyglucose
l-Man: l-mannose
ARM: antibody-recruiting molecule
αGal: galactose-α1,3-galactose
TBM: tumor-binding molecule
DNP: 2,4-dinitrophenol
cARM: covalent antibody-recruiting molecule
l-Col: l-colitose
RBL: Rha-binding lectin
TL: tachylectin
APEX2: ascorbate peroxidase
FT: fucosyltransferase
LacNAc: *N*-acetyl-d-lactosamine
FACS: fluorescence-activated cell sorting
LiGA: liquid glycan array
LiLa: liquid lectin array
MS/MS-IR: mass spectroscopy and infrared ion spectroscopy
CID: collision-induced dissociation
